# Psychological factors are associated with the outcome of physiotherapy for people with shoulder pain: a multicentre longitudinal cohort study

**DOI:** 10.1136/bjsports-2016-096084

**Published:** 2016-07-21

**Authors:** Rachel Chester, Christina Jerosch-Herold, Jeremy Lewis, Lee Shepstone

**Affiliations:** 1 Faculty of Medicine and Health Sciences, School of Health Sciences, University of East Anglia, Norwich, Norfolk, UK; 2 Physiotherapy Department, Norfolk and Norwich University Hospital, Norwich, Norfolk, UK; 3 Department of Allied Health Professions, School of Health and Social Work, University of Hertfordshire, Hatfield, UK; 4 Faculty of Medicine and Health Sciences, Norwich Medical School, University of East Anglia, Norwich, Norfolk, UK

**Keywords:** Shoulder, Physiotherapy, Assessment, Psychology

## Abstract

**Background/aim:**

Shoulder pain is a major musculoskeletal problem. We aimed to identify which baseline patient and clinical characteristics are associated with a better outcome, 6 weeks and 6 months after starting a course of physiotherapy for shoulder pain.

**Methods:**

1030 patients aged ≥18 years referred to physiotherapy for the management of musculoskeletal shoulder pain were recruited and provided baseline data. 840 (82%) provided outcome data at 6 weeks and 811 (79%) at 6 months. 71 putative prognostic factors were collected at baseline. Outcomes were the Shoulder Pain and Disability Index (SPADI) and Quick Disability of the Arm, Shoulder and Hand Questionnaire. Multivariable linear regression was used to analyse prognostic factors associated with outcome.

**Results:**

Parameter estimates (β) are presented for the untransformed SPADI at 6 months, a negative value indicating less pain and disability. 4 factors were associated with better outcomes for both measures and time points: lower baseline disability (β=−0.32, 95% CI −0.23 to −0.40), patient expectation of ‘complete recovery’ compared to ‘slight improvement’ as ‘a result of physiotherapy’ (β=−12.43, 95% CI −8.20 to −16.67), higher pain self-efficacy (β=−0.36, 95% CI −0.50 to −0.22) and lower pain severity at rest (β=−1.89, 95% CI −1.26 to −2.51).

**Conclusions:**

Psychological factors were consistently associated with patient-rated outcome, whereas clinical examination findings associated with a specific structural diagnosis were not. When assessing people with musculoskeletal shoulder pain and considering referral to physiotherapy services, psychosocial and medical information should be considered.

**Study registration:**

Protocol published at http://www.biomedcentral.com/1471-2474/14/192.

## Introduction

First episode of shoulder pain accounts for nearly 1.5% of visits to the general practitioner (GP) annually within the UK.^1^ ^2^ This represents a considerable burden, with up to 48% of patients visiting their GP more than once due to the ongoing shoulder pain.^1^ ^2^ As most treatment options have equivocal effectiveness,^3^ ^4^ doctors and patients face uncertainty when deciding on the most appropriate form of management. Non-surgical treatment is typically the first-line management,^4^ ^5^ with the majority of referrals directed to physiotherapy services.^1^ ^6^ Although this represents up to 14% of referrals to UK outpatient physiotherapy services each year,^7^ there is no clear and current guidance as to (1) which patients with shoulder problems should be referred and (2) which clinical and psychosocial factors are associated with better or worse outcomes.

Prognostic factors associated with the outcome of physiotherapy for shoulder pain are unclear, and currently cannot support clinical decision-making.^8^ Our previous review highlighted the need for an adequately sized study to investigate a wider range of biopsychosocial variables as potential prognostic factors. This is needed as the optimal treatment for shoulder pain remains unclear.^9^

The objective of this multicentre longitudinal cohort study was to identity which patient and clinical characteristics, commonly assessed at the first physiotherapy appointment, were associated with patient-rated shoulder pain and function, at 6 weeks and 6 months.

## Methods

### Study design and participants

The study protocol for this multicentre longitudinal cohort study has been published in detail elsewhere,^10^ and is summarised here. Patients referred to physiotherapy for the management of musculoskeletal shoulder pain at 11 NHS trusts and social enterprises in the East of England were recruited to the study between November 2011 and October 2013. Participating physiotherapy departments were located within primary and secondary care.

A postal invitation was sent to the patients whose referral to physiotherapy indicated that they may be eligible for the study. Patients were eligible if they were aged 18 years or older and described shoulder or arm pain aggravated by shoulder movements. Patients with significant reproduction of shoulder pain on spinal movement, or greater reproduction on spinal movement compared to shoulder movement, were excluded from the study. Patients with the following aetiology for shoulder pain were excluded: radiculopathy, postsurgery, postfracture, posttraumatic dislocation or systemic source. Participants provided written informed consent at their first physiotherapy appointment. Patients who decided not to participate in the study, or did not respond to later follow-ups, were not required to provide a reason.

### Data collection

Data for 71 putative prognostic factors, determined and defined a priori, were collected from each participant and their physiotherapist. Summary baseline characteristics are presented in table 1. See online supplementary files S1–3 for the full list of putative prognostic factors. These were identified and selected from our literature review of previous studies of prognostic factors for the physiotherapy management of shoulder pain,^8^ prognostic factors documented for other management approaches^11^ or musculoskeletal areas and workshops with participating clinicians, and patient and public representatives during protocol development. There was no convincing evidence from previous studies that psychological measures were associated with outcome for our specific population.^8^ This was in contrast to studies which included populations with low back pain^12 13^ or general upper quadrant and/or cervical pain.^14^ ^15^ Patient expectation was included as it is associated with outcome in the wider medical field.^16^ Measurement tools were selected from a broad range of sources,^17–19^ while minimising excessive participant and physiotherapist burden.

**Table 1:** Selected summary baseline characteristics of participants (n=1030)

Factor	Category	Mean (SD)	Number (%)
Demographics, including self-rated pain and disability			
Age (years)		57 (15)	
Sex (male)			455 (44)
Index of multiple deprivation		15 (10)	
Baseline SPADI		48 (22)	
Baseline QuickDASH		38 (18)	
Participants' pain beliefs, experience and expectations			
Pain self-efficacy questionnaire^18^ 0–60, 60=greater efficacy		44 (13)	
General health			
Body mass index, mean (SD)		27 (5)	
Anxiety and depression in the previous 7 days	No		657 (64)
	Moderately		347 (34)
	Extremely		26 (<3)
Musculoskeletal pain outside the affected upper quadrant: (see online supplementary file S1 for further detail)	None		787 (76)
	One additional site		185 (18)
	≥2 additional sites		58 (6)
Additional health problems (see online supplementary file S1 for further detail)	None		551 (53)
	One additional		298 (29)
	≥2 additional		181 (18)
Lifestyle			
Smoker (cigarettes, cigars or pipe)	Yes		129 (13)
	Stopped for the last 10 years		117 (11)
	Stopped >10 years		261 (25)
	No, never		523 (51)
Highest level of leisure time exercise intensity in a typical week* Godin leisure time exercise questionnaire^44^	Strenuous		222 (22)
	Moderate		333 (32)
	Mild		348 (34)
	None		124 (12)
Current frequency of pain medication	None		258 (25)
	Very occasional		360 (35)
	Most days and/or nights		217 (21)
	Every day and/or night		195 (19)
Work			
Currently off work due to shoulder pain	Yes		18 (<2)
Time off work due to shoulder pain in last year	Yes	127 (12)	
Nature of employment	Employed/education		599 (58)
	Retired		364 (36)
	Currently not working		62 (6)
Shoulder symptoms			
Severity of shoulder pain at rest (0–10 numerical rating scale)		3 (3)	
Duration of shoulder pain (months)		14 (28)	

(see online supplementary files S1–3 for the full list and further details of putative prognostic factors investigated).

*Unit of measurement used for data analysis modified after data collection.

SPADI, Shoulder Pain and Disability Index; QuickDASH, Quick Disability of the Arm, Shoulder and Hand Questionnaire.

10.1136/bjsports-2016-096084.supp1 Supplementary file

10.1136/bjsports-2016-096084.supp2 Supplementary file

10.1136/bjsports-2016-096084.supp3 Supplementary file

Prior to the first physiotherapy appointment, participants completed a bespoke questionnaire. At the first appointment, using standardised clinical data forms, physiotherapists recorded the history of the participant's shoulder problem and clinical examination findings. At discharge, physiotherapists recorded details of treatment and attendance on a standardised clinical data form. The delivery and content of treatment were unaffected by participation in the study.

Participants were sent a postal follow-up questionnaire, 6 weeks and 6 months after starting their course of physiotherapy. This included two validated patient-reported outcome measures also collected at baseline: the Shoulder Pain and Disability Index (SPADI)^20^ ^21^ and the Quick Disability of the Arm, Shoulder and Hand Questionnaire (QuickDASH).^22^ Scores for these outcomes are expressed as a percentage, where zero represents no pain or disability and 100% represents maximum pain and disability. The participants did not have access to the responses they provided at baseline or at 6-week follow-up. Participants were also asked to return details of exercise adherence, recorded using a paper diary supplied at their first physiotherapy appointment, and provide details of any other interventions they may have received. To maximise response rates, non-responders at each time point were sent a maximum of two reminders to return their questionnaires.

### Statistical analysis

Using the approach suggested by Lipsitz and Parzen,^23^ based on analysis with a general linear model, 780 participants provided 90% statistical power to detect an effect size of <0.25 SDs adjusted for other variables with a coefficient of determination (ie, R^2^) with the outcome of up to 0.5. Therefore, 1000 participants were allowed for up to 22% loss to follow-up and this was the target sample size. All the participants providing outcome data at 6 weeks and/or 6 months were included in the analysis for that time point, whether or not they had completed their course of physiotherapy. Missing data at one time point were not imputed from other time points. All statistical analyses were carried out in STATA V.12.

For each outcome (the SPADI and QuickDASH) and time point (6 weeks and 6 months), general linear models were constructed. These were constructed with and without the inclusion of baseline SPADI and QuickDASH values. Also, as the residuals were not normally distributed, a logit transformation was applied to the outcomes and the transformed data were also modelled. For ease of clinical interpretation, the parameter estimates presented here are for the untransformed models.

Initially, all putative prognostic variables were singularly entered into simple linear regression models. Those with a statistically significant relationship with the outcome, at the 10% level, were entered into a multivariable linear regression model. A stepwise selection process, based on change in scaled deviance, was then applied. The explanatory variable with the least significant p Value was removed. This process was continued until all the remaining variables had regression coefficients significant at the 5% level (ie, p<0.05). Each variable removed on backward elimination was then individually re-entered and remained in the model if it attained a statistical significance of 5%.

This process was carried out for each of the nine subgroups of variables indicated in online supplementary files S1–3. The explanatory variables in these models were then forwarded for inclusion in a final model. Factors that were not significant in group models, but had been associated with outcome at other time points, in other studies, or were considered key prognostic factors associated with outcome for other musculoskeletal regions, were also entered. The process of backward elimination and forward selection was repeated as above. The adjusted coefficient of determination (R^2^), a proportional measure indicating the amount of variation in outcome explained by the models, is presented.

Logistic regression was used to compare consenters with non-consenters, and the characteristics of participants who provided and did not provide outcome data. Adjusted odds ratio (OR) with 95% CIs are presented for statistically significant differences at p≤0.05.

## Results

**Figure 1:**
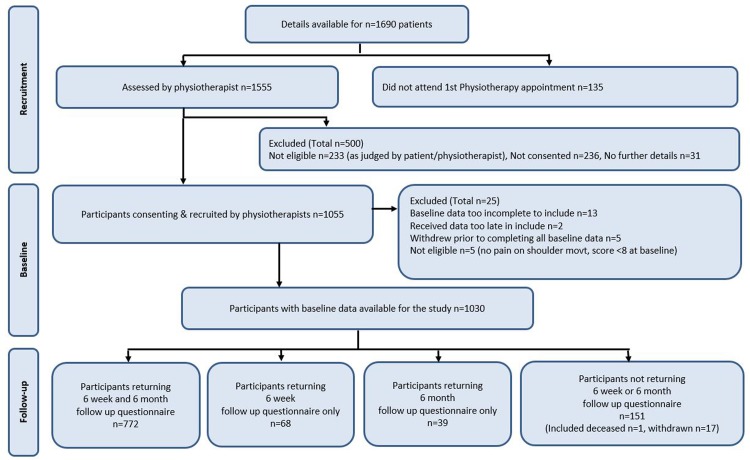
STROBE flow diagram. Participant recruitment and follow-up.

One thousand and fifty-five participants were recruited and consented to be in the study, 1030 of whom were eligible, provided adequate baseline data and were included in the study. Details of patient characteristics and baseline measures investigated as potential prognostic factors are presented under their respective subgroup headings in table 1 and online supplementary files S1–3. There were no factors at baseline for which >2% of data were missing. Eight-hundred and forty (82%) participants provided outcome data at 6 weeks, 811 (79%) at 6 months and 772 (75%) at 6 weeks and 6 months. See STROBE flow diagram in figure 1.

There was no significant difference in mean age or sex between consenters (57 years, SD=15, 44% male) and non-consenters (56 years, SD=16, 47% male). Having provided baseline data, 17 participants withdrew from the study, 1 died and 151 did not return their questionnaire or respond to reminders at either follow-up point. Participants who provided outcome data at 6 weeks and 6 months differed at baseline from those who did not (table 2). They were older, reported greater pain self-efficacy, were almost twice as likely to do some form of leisure time activity, three times as likely to have completed their course of physiotherapy and were less likely to have non-attendance.

**Table 2:** Differences between participants who provided outcome data (n=772) at 6 weeks and 6 months and those who did not (n=258)

Prognostic factor	Provided full outcome data	Mean (SD)	OR	95% CI	p Value
Age	No	48.73 (15.02)			
	Yes	58.92 (13.79)	1.06	1.04 to 1.07	<0.001
Pain self-efficacy	No	41.00 (13.50)			
	Yes	44.84 (12.54)	1.02	1.01 to 1.04	0.001
Number of appointments attended	No	4.15 (2.81)			
	Yes	5.01 (2.74)	1.11	1.04 to 1.18	<0.001
Number of non-attendances	No	0.80 (1.06)			
	Yes	0.45 (0.99)	0.83	0.71 to 0.97	0.018
		**Percentage**			
Most strenuous weekly exercise		*No exercise (no=16, yes=11)	0		
		Mild exercise (no=24, yes=36)	1.96	1.12 to 3.41	0.019
		Moderate exercise (no=30, yes=33)	1.89	1.09 to 3.28	0.023
		Strenuous exercise (no=30, yes=20)	1.72	0.96 to 3.13	0.069
Course of Physiotherapy completed	No	61%			
	Yes	87%	3.06	2.09 to 4.49	<0.001

OR presented for participants who provided full outcome data compared to those not providing full outcome data. no, did not provide full outcome data at both follow up points. yes, provided full outcome data at both follow up points.

*‘No exercise’ is the reference category for the analysis of ORs for the most strenuous weekly exercise.

After multivariable linear regression, each model contained between 7 and 12 prognostic factors explaining between 0.34 and 0.48 of the variance at 6 weeks and between 0.30 and 0.43 of the variance at 6 months. Parameter estimates for the non-transformed SPADI at 6 months are presented in table 3. A negative parameter estimate refers to a decrease in SPADI (less pain and disability), and a positive parameter estimate refers to an increase in SPADI (more pain and disability). A post hoc analysis indicated that the addition of treatment factors, clinical setting and seniority of the assessing physiotherapist did not change the association of prognostic factors with outcome in any of the models, neither did the inclusion of a random-effect term to quantify variation between physiotherapists.

**Table 3:** Multivariable linear regression for SPADI at 6-month follow-up: n=804, adjusted R^2^=0.31 (baseline SPADI and QuickDASH not included in the model)

Independent predictor	Subgroup comparisons	Parameter estimate	95% CI	p Value
Patient expectation of change	Completely recover	0		
	Much improve	5.21	1.80 to 8.61	0.003
	Slightly improve	12.43	8.20 to 16.67	<0.001
	No change/worse	−0.94	−8.53 to 6.66	0.809
Pain self-efficacy		−0.36	−0.50 to −0.22	<0.001
Number of additional health problems	None	0		
	One	3.52	0.30 to 6.75	0.032
	Two or more	6.62	1.48 to 9.75	0.008
Anxiety or depression	None	0		
	Moderate	2.19	−0.99 to 5.37	0.177
	Extreme	12.02	1.49 to 22.56	0.025
Frequency of pain medication	None/occasional	0		
	Most/every day/night	3.85	0.61 to 7.09	0.020
Most strenuous exercise	None	0		
	Mild	−5.53	−10.32 to −0.74	0.024
	Moderate	−8.98	−13.86 to −4.11	<0.001
	Strenuous	−6.82	−12.17 to −1.47	0.013
Difference between passive and active abduction		0.07	0.004 to 0.142	0.038
Change during scapular facilitation	Yes	0		
	No	4.93	2.13 to 7.74	0.001
Severity of pain at rest		1.89	1.26 to 2.51	<0.001
Duration of shoulder symptoms (weeks)		0.02	0.01 to 0.03	0.002
Paraesthesia in the arm	No	0		
	Yes	−10.08	−18.73 to −1.43	0.022
Current employment status	Employed	0		
	Retired	2.96	0.10 to 6.02	0.058
	Unemployed	14.30	7.72 to 20.87	<0.001

The first row of prognostic factors with categorical data is the reference or comparator category.

SPADI, Shoulder Pain and Disability Index; QuickDASH, Quick Disability of the Arm, Shoulder and Hand Questionnaire.

The factors relating to outcome for either the SPADI or QuickDASH at 6 weeks and 6 months are summarised in table 4. Four factors were associated with a better outcome for the SPADI and QuickDASH at both time points: lower baseline pain and disability measured by the corresponding outcome measure, patient expectation of a ‘complete recovery’ as ‘a result of physiotherapy treatment’ in comparison to ‘slight improvement’, higher pain self-efficacy and lower pain severity at rest. In addition, with one exception (the SPADI at 6-week follow-up), being in current employment or education was associated with a better outcome than not being in current employment or education.

**Table 4:** Factors predicting a worse outcome at 6 weeks and 6 months (in three or all of the four models for each outcome and time point)

	At 6 weeks and 6 months	At 6 weeks only	At 6 months only
SPADI and QuickDASH	Higher baseline pain and disability measured by corresponding outcome measure. Patient expectation of ‘slight improvement’ rather than ‘complete recovery’ as ‘a result of physiotherapy treatment’. Lower pain self-efficacy. Not being in employment due to redundancy, unemployment or disability compared with being in employment or education (exception: SPADI at 6 weeks). Higher pain severity at rest.	Previous compared to no previous major operation (shoulder surgery excluded). The presence, compared to the absence, of pain in the opposite upper quadrant.	One and in particular two or more additional health problems compared to none. Patient expectation of ‘much improvement’ rather than ‘complete recovery’ as ‘a result of physiotherapy treatment’. Longer duration of shoulder symptoms. No change compared to change in shoulder pain/range during manual facilitation of the scapula around the chest wall during arm elevation.
SPADI only		Patient expectation of ‘no change’ rather than ‘complete recovery’ as ‘a result of physiotherapy treatment’. Reduced range of active shoulder abduction.	Most strenuous weekly exercise classified as ‘none’ compared to ‘moderate’. Increasing difference between the range of active and passive shoulder abduction.
QuickDASH only		Being female rather than male.	Both shoulders affected or patient stated ‘ambidextrous’ in comparison to only non-dominant shoulder affected.

SPADI, Shoulder Pain and Disability Index; QuickDASH, Quick Disability of the Arm, Shoulder and Hand Questionnaire.

At 6 weeks only, a better outcome for both measures was associated with the absence, compared to the presence, of pain in the opposite upper quadrant (SPADI, β=−8.60, 95% CI −4.33 to −12.87) and no previous, compared to a previous, major operation (shoulder surgery excluded) (SPADI, β=−8.11, 95% CI −3.66 to −12.56).

At 6 months only, a better outcome for both measures was associated with a shorter duration of symptoms, no additional health problems compared to one and in particular two or more and a reduction in pain or increase in the range of shoulder elevation with manual facilitation of the scapula during elevation of the arm (table 4).^24^ ^25^

A number of clinical examination findings, commonly associated with specific shoulder disorders, were not associated with outcome. For example, (1) restricted passive external rotation, due to pain or stiffness, indicative of a frozen shoulder,^26^ and (2) an external rotation lag,^27^ indicative of a rotator cuff tear, were not associated with outcome. Neck pain or pain on movement of the cervical spine, during the clinical examination, was not associated with outcome.

## Discussion

### Summary of main findings

Ten prognostic factors were consistently associated with the SPADI and QuickDASH at one or both time point(s). This is the first known study to investigate the association of five of these factors with the outcome of physiotherapy management for shoulder pain. These five factors that were associated with a better outcome include: (1) patient expectation of ‘complete recovery’ compared to a ‘slight improvement’ as ‘a result of physiotherapy treatment’, (2) lower pain severity specifically at rest, (3) the absence of a previous major operation (shoulder surgery excluded), (4) the absence of pain in the opposite upper quadrant and (5) change in pain or range of shoulder elevation with manual facilitation of the scapula during elevation of the arm.

### Strengths and limitations

For the physiotherapy management of shoulder pain, this multicentre prospective study of over 1000 participants is the first to include a range of biopsychosocial factors with a validated psychological measure and clinical examination findings with predefined standard operating procedures. It is the first study of this magnitude to include patients who start but do not complete their course of physiotherapy. The results are generalisable to the wide range of patients and presentations of shoulder pain commonly seen within physiotherapy practices within primary and secondary care.

As part of their treatment, 99% of participants were required to carry out a home exercise programme prescribed by their physiotherapist. Prognostic factors associated with outcome for this active intervention on behalf of the patient may differ from those identified for more passive management options such as surgery or other conservative options.

The limitations associated with this study must be acknowledged when interpreting the results. Only prognostic factors significant in three of four models for each outcome and time point were presented. Factors remaining in the models for the transformed and non-transformed outcome data were generally the same with no discrepancies in the direction of association; we presented parameter estimates for non-transformed outcome data to facilitate clinical interpretation.

There was a differential non-response at follow-up. There was a greater likelihood of missing outcome data for younger participants and those not partaking in leisure time physical activity. This may have decreased the power of the study to detect a more consistent association of these factors with outcome and represent a potential source of bias. Similarly, low numbers of patients with extreme anxiety and depression participated in the study, which decreased the power of the study to detect an association between anxiety and depression and outcome.

### Comparison with other studies

Baseline disability and pain self-efficacy have been investigated in previous studies: in our study, higher disability at baseline was associated with higher disability at follow-up, which is consistent with three previous studies.^28–30^ Pain self-efficacy is the extent or strength of the patient's belief in their ability to complete tasks and perform certain behaviours^31^ despite their pain. Pain self-efficacy was investigated in one previous study, which found no significant association with the SPADI at 1 year.^32^ The study participants were part of a randomised controlled trial, and physiotherapy consisted primarily of supervised exercises or radial extracorporeal shockwave therapy.^33^ In our study, 99% of the participants were given a home exercise programme, often as a primary aspect of their treatment. The level of pain self-efficacy required to learn, undertake and effectively implement a prescribed home exercise programme may be greater than that required for the supervised exercises or therapist-administered interventions and may explain the significant association with outcome observed in our study.

Not being in employment due to redundancy, unemployment or disability was significantly associated with poorer outcomes for the QuickDASH at 6 weeks and for both measures at 6 months. Heterogeneity on a number of levels, in particular differences in the case definition of comparator groups, inhibits direct comparison with the two additional studies for which this has been investigated,^32^ ^34^ none of which report a significant association with outcome. However, long-term disability and unemployment have been demonstrated as prognostic factors for a poor outcome for the management of low back pain in primary care.^35^ ^36^

Symptom duration and comorbidities have been investigated in previous studies. Of five studies investigating an association between symptom duration and end point scores, two demonstrated that a longer symptom duration was associated with a poorer outcome,^29^ ^37^ and three studies demonstrated no association.^28^ ^30^ ^32^ An inconsistent association between symptom duration and outcome has been reported for other forms of conservative management at the shoulder.^11^ Two previous studies investigated the association between comorbidities and end point scores, neither of which demonstrated a significant association.^28^ ^29^ Our results suggest that a previous major operation, which was not specifically included in previous studies, is an important factor to include within the list of comorbidities.

The most consistent factor associated with outcome in our study, patient expectation of recovery, is recognised as a prognostic factor for a wide range of other health problems.^16^ It is unknown whether treatment response is a consequence of patient expectation, the subsequent physiological mechanisms, whether psychologically and behaviourally, participants expecting a recovery may be more observant of positive improvements in their symptoms or whether patients are simply good at predicting their outcome.^16^ ^38^ This study suggests that a positive patient expectation of recovery as a result of physiotherapy should be reinforced by clinicians.

Shoulder pain severity at rest was another consistent factor associated with outcome. Shoulder pain severity has been investigated as a prognostic factor in four previous studies,^28^ ^34^ ^37^ ^39^ none of which demonstrated an association with outcome. However, ours is the only study specifically measuring resting pain. Given that this is predictive of a poorer outcome, more targeted multidisciplinary input may be required for patients with resting pain.

This is the first study to investigate the prognostic value of symptom modification with scapular facilitation techniques.^24^ ^25^ The underlying mechanisms responsible for a change in symptoms are unknown^24^ as abnormal scapular movement and posture are not consistently associated with shoulder pain or any specific structural pathology.^40^ The value of this assessment technique may be that positive findings indicate a mechanical component to the symptoms, which may respond to treatment techniques used by physiotherapists.

### Implications for practice and research

This study provides evidence to support the NICE^5^ recommendation of a general approach to the assessment of shoulder pain. The International Classification of Functioning Disability and Health^41^ states that body structure and function, activity and participation and personal and environmental factors are integral to health for all individuals. Our study demonstrated that prognostic factors associated with outcome cover a broad range. Psychological factors were consistently associated with outcome. In comparison, clinical examination findings, suggestive of a structural diagnosis, were not consistently associated with the outcome of physiotherapy management. This is important as clinicians assessing and managing shoulder pain are taught to perform structural differentiation procedures, which can account for a substantial component of the patient examination. The findings of this investigation indicate that when doctors and their patients are deciding whether or not to pursue physiotherapy as a potentially effective management option, psychosocial and biomedical information should be considered.

Physiotherapists stated their expectation of change using a similar seven-point Likert scale to that completed by their patient. However, physiotherapists completed the scale following rather than prior to the assessment. The physiotherapists' expectation of change was not associated with outcome. Nor was it associated with the patient's expectation of recovery. This suggests that physiotherapists' predictions of how well a patient will respond to treatment cannot be relied on. A more formalised approach is required.

We suggest that baseline psychological factors such as pain self-efficacy and patient expectation should be formally assessed using standardised measures. These psychological factors have a prognostic value, not just at extreme values, but also throughout a range of possible values and responses. For example, there was a statistically significant difference in outcome at 6 months between patients rating expectation of recovery on adjacent points of the seven-point Likert scale: patients who expected a ‘complete recovery’ had better outcomes compared to those who expected to ‘much improve’.

We did not investigate the effect of different treatment approaches. However, poorer outcomes in individuals with certain factors might suggest that addressing these factors could result in better outcomes. We, therefore, encourage doctors and physiotherapists to consider integrating psychological interventions within their everyday practice to manage all patients with shoulder pain. For example, motivational interventions used by physiotherapists can improve self-efficacy for a range of musculoskeletal and medical problems,^42^ and brief psychological interventions implemented in GP surgeries can promote physical activity.^43^ Given the consistent association between a positive expectation of change and a positive outcome in our study, and the potential influence of physicians' beliefs on patient expectation, we encourage physicians and physiotherapists to reinforce a positive expectation when referring patients to physiotherapy.

A multidisciplinary team may enhance outcomes for some patients. For patients with lower self-efficacy or only a slight expectation of recovery, who have not responded to psychological interventions within the GP or physiotherapist's practice, we suggest early involvement of psychological experts. For patients complaining of resting shoulder pain or pain associated with other comorbidities, particularly previous major surgery, we suggest treatments aimed at pain relief. Given the magnitude of association between unemployment and a poorer outcome from physiotherapy, we also encourage physicians to consider referral to employment support services for patients unable to work due to their shoulder pain. We also suggest that patients not engaging in leisure time physical activity are encouraged to do so and those taking part are encouraged to remain doing so, in some capacity.

Future research is needed to externally validate a clinical prediction model based on the prognostic factors identified in this study including the question of which prognostic factors can be successfully modified with treatment delivered by physiotherapists and if this improves outcome. Future research should identify which factors predict how subgroups of patients are likely to benefit from individual treatments offered by physiotherapists. This would form the basis of personalised or stratified healthcare for the treatment of shoulder pain.

**What are the findings?**

- Higher patient expectation of recovery as a result of physiotherapy, higher pain self-efficacy, lower pain severity at rest, and for patients not retired, being in employment or education were associated with a better outcome.
- Clinical examination findings suggestive of a structural diagnosis were inconsistently associated with outcome.
- Physiotherapists’ predictions of how well a patient will respond to treatment cannot be relied on. A more formalised approach is required.
- Psychosocial in addition to biomedical information should be formally assessed and feed into decision-making about management options.

**How might it impact on clinical practice in the near future?**

- Physicians referring patients to physiotherapy should reinforce a positive expectation of recovery as a result of physiotherapy treatment.
- Psychological factors, such as patient expectation and pain self-efficacy should be formally assessed using standardised measures.
- Patients with resting pain and/or pain arising from other comorbidities may be provided and guided on appropriate pain medication or other pain-relieving treatments prior to or at the same time as referral to physiotherapy.
- A multidisciplinary approach should be considered for patients with more extreme psychological responses associated with a poorer outcome, resting shoulder pain not responding to medication provided by their physician, and patients not currently employed or in education but of working age.
