# Supplementary material for: Psychological factors are associated with the outcome of physiotherapy for people with shoulder pain: a multicentre longitudinal cohort study
Source: Br J Sports Med. 2016 Jul 21;52(4):269–75. doi: 10.1136/bjsports-2016-096084 (PMC5867439; doi:10.1136/bjsports-2016-096084)
Supplement: Supplementary file [file bjsports-2016-096084supp001.pdf]

Supplementary file 1: Participants baseline characteristics (n=1030)

Factor	Category	Mean (SD)	Number (%)
**Demographics, including self-rated pain and disability**			
Age (years)		57 (15)	
Sex (male)			455 (44)
Index of multiple deprivation		15 (10)	
Baseline SPADI		48 (22)	22
Baseline QuickDASH		38 (18)	18
**Participants' pain beliefs, experience and expectations**			
Pain Self-Efficacy Questionnaire[23] 0-60, 60=greater efficacy,		44 (13)	13
Physiotherapy for previous shoulder problems helpful	Yes		137 (13)
	In part		77 (8)
	No		35 (3)
	Not applicable		781 (76)
Physiotherapy for other health problems helpful	Yes		372 (36)
	In part		130 (13)
	No		54 (5)
	Not applicable		474 (46)
Patient perceived importance of physiotherapy in helping to gain maximum possible recovery (1-5 Likert scale, 1="Not at all" & 5= "Extremely")	5		505 (49)
	4		267 (26)
	3		196 (19)
	2		45 (4)
	1		11 (1)
Patient perceived confidence in their ability to manage shoulder symptoms with their physiotherapist (1-5 Likert scale, 1="Not at all" & 5= "Extremely")	5		465 (45)
	4		317 (31)
	3		193 (19)
	2		41 (4)
	1		8 (1)
Patient readiness to work with the physiotherapist and do any prescribed exercises at home (1-5 Likert scale, 1="Not at all" & 5= "Extremely")	5		824 (80)
	4		166 (160)
	3		32 (30)
	2 or 1		<5 (10)
Participant's response to "How much do you expect your shoulder problem to change as a result of physiotherapy treatment?" (7 point Likert scale)	Completely recover		246 (24)
	Much improve		539 (52)
	Slightly improve		197 (19)
	No change		38 (4)
	Worse (3 options)		9 (1)
Physiotherapist's response to "How much do you expect this patient's current shoulder problem to change as a result of this course of physiotherapy treatment?" (7 point Likert scale)	Completely recover		112 (11)
	Much improve		571 (55)
	Slightly improve		322 (31)
	No change		21 (2)
	Worse (3 options)		3 (<1)
**General Health**			
Body mass index, Mean (SD)		27 (5)	
Anxiety and depression in the previous seven days	No		657 (64
	Moderately		347 (34
	Extremely		26 (<3)
Difficulty sleeping even if don't have shoulder pain	No		564 (55)

	Sometimes		385 (37)
	Most nights		81 (8)
Musculoskeletal pain outside the affected upper quadrant:	No		787 (76)
Includes opposite upper quadrant 110 (11%), trunk/abdomen 61 (6%), head/neck 107 (10%), lower limbs 38 (4%)	One additional site		185 (18)
	≥2 additional sites		58 (65)
Additional health problems.	No		551 (53)
Includes heart 105 (10%), mental health 89 (9%), major operation 95 (9%), respiratory 93 (9%), diabetes 87 (8%), joint replacement 54 (5%), other 92 (9%). Each less than 50 (5%): epilepsy, cancer. rheumatic condition (shoulder unaffected), uncontrolled high BP	One additional		298 (29)
	≥2 additional		181 (18)
**Lifestyle**			
Smoker (cigarettes, cigars or pipe)	Yes		129 (13)
	Stopped last 10 years		117 (11)
	Stopped > 10 years		261 (25)
	No, never		523 (51)
Highest level of leisure time exercise intensity in a typical week* Godin leisure time exercise questionnaire[24]	Strenuous		222 (22)
	Moderate		333 (33)
	Mild		348 (34)
	None		124 (12)
Frequency of regular activity long enough to build up a sweat	Often		211 (20)
	Sometimes		418 (41)
	Never/rarely		399 (39)
Current frequency of pain medication	None		258 (25
	Very occasional		360 (35
	Most days &/or nights		217 (21
	Every day &/or night		195 (19
**Work**			
Currently off work due to shoulder pain	Yes		18 (<2)
Time off work due to shoulder pain in last year (days)		127 (12)	
Time off work due to other health probs. in last 6 months (days)		182 (18)	
Nature of employment	Employed/Education		599 (58)
	Retired		364 (36)
	Currently not working		62 (6)
Type of work or regular activity	Overhead	358	358 (35)
	Sedentary	391	391 (38)
	Heavy manual	327	327 (32)

*Unit of measurement used for data analysis modified after data collection.
