# Supplementary material for: Psychological factors are associated with the outcome of physiotherapy for people with shoulder pain: a multicentre longitudinal cohort study
Source: Br J Sports Med. 2016 Jul 21;52(4):269–75. doi: 10.1136/bjsports-2016-096084 (PMC5867439; doi:10.1136/bjsports-2016-096084)
Supplement: Supplementary file [file bjsports-2016-096084supp002.pdf]

Supplementary file 2: Participants shoulder symptoms (n=1030)

Factor	Category	Mean (SD)	Frequency (percent)
Side affected	Dominant		557 (54)
	Non dominant		381 (37)
	Bilateral		74 (7)
	Ambidextrous		18 (2)
Cause of onset	No clear reason		523 (51)
	Trauma		229 (22)
	Overuse or repetition		167 (16)
	Unusual activity		105 (10)
Timing of onset	Insidious		541 (53)
	Sudden		450 (44)
	Episodic		37 (3)
History of past shoulder pain	Yes		415 (44)
Number of painful regions within affected upper quadrant		3 (1)	
Painful areas and frequency of symptoms: anterior shoulder 737 (72%), posterior shoulder 328 (32%), lateral shoulder 484 (47%), superior shoulder including acromio clavocular joint (epaulette) 338 (33%), scapula 181 (18%), chest and thorax 99 (10%), brachial 533 (52%), below elbow 157 (15%), paraesthesia in hand 100 (10%) or proximal to hand 26 (<3%).			
