# Supplementary material for: Psychological factors are associated with the outcome of physiotherapy for people with shoulder pain: a multicentre longitudinal cohort study
Source: Br J Sports Med. 2016 Jul 21;52(4):269–75. doi: 10.1136/bjsports-2016-096084 (PMC5867439; doi:10.1136/bjsports-2016-096084)
Supplement: Supplementary file [file bjsports-2016-096084supp003.pdf]

Supplementary file 3: Participant clinical examination findings (n=1030)

Clinical examination of shoulder joints	Mean (SD)	Frequency (percent)
**Range of shoulder movement (Degrees)***		
Active flexion	126 (34)	
Passive flexion	139 (30)	
Active abduction	114	
Passive abduction	126	
Passive external rotation	51	
**Primary factor restricting each shoulder movement**		
**Active flexion**		128 (12)
Normal		188 (16)
Stiffness		681 (66)
Pain		27 (3)
Weakness		6 (<1)
Apprehension		
**Passive flexion**		189 (18)
Normal		231 (22)
Stiffness		594 (58)
Pain		14 (1)
Apprehension		
**Active abduction**		99 (10)
Normal		160 (16)
Stiffness		736 (71)
Pain		29 (3)
Weakness		6 (<1)
Apprehension		
**Passive abduction**		174 (17)
Normal		193(19)
Stiffness		646(63)
Pain		14(1)
Apprehension		
**Passive external rotation**		305 (30)
Normal		257 (25)
Stiffness		457 (44)
Pain		7 (<1)
Apprehension		
**Clinical examination, contractile**		
Difference between active and passive flexion (degrees)	13 (18)	
Difference between active and passive abduction (degrees)	13 (3)	
Presence of an "external rotation lag"[29]		88 (9)
**Force of shoulder movement (Newton)****		
Abduction	43 (28)	
External rotation	47 (30)	
**Percentage force compared to unaffected side**		
Abduction	71 (34)	
External rotation	79 (30)	

Clinical examination, other	Frequency	Percent
**Arm pain on movements of the cervical spine**		
None	917	90
Yes, but no reproduction of shoulder pain	97	9
Yes, some minor reproduction of shoulder pain***	15	1
**Positive change in pain or range of movement during manual facilitation of scapula during active abduction**		
Complete resolution of symptoms or restoration of movement	122	12
Some change, (>30%, but not complete resolution)	426	41
No change	482	47
**Primary clinical problem stated by physiotherapist (frequency and percentage)**		
Pain	763	74
Stiffness	209	20
Weakness	85	8
Instability	15	1

Legend: Data refers to affected shoulder only unless otherwise indicated.

*Range of shoulder movement measured using a goniometer.

**Force of shoulder movement measured using a hand held myometer (Mecmesin basic digital or analogue force gauge)

*** Patients with significant reproduction of shoulder pain on movements of the cervical spine were excluded as were patients with a greater reproduction of shoulder pain on movement of the cervical spine in comparison to the shoulder.
